# Systematic review of translational insights: Neuromodulation in animal models for Diabetic Peripheral Neuropathy

**DOI:** 10.1371/journal.pone.0308556

**Published:** 2024-08-08

**Authors:** Rahul Mittal, Keelin McKenna, Grant Keith, Evan McKenna, Rahul Sinha, Joana R. N. Lemos, Khemraj Hirani

**Affiliations:** 1 Diabetes Research Institute, University of Miami Miller School of Medicine, Miami, Florida, United States of America; 2 Herbert Wertheim College of Medicine, Florida International University, Miami, Florida, United States of America; 3 School of Medicine and Public Health, University of Wisconsin, Madison, Wisconsin, United States of America; NYU Grossman Long Island School of Medicine, UNITED STATES OF AMERICA

## Abstract

Diabetic Peripheral Neuropathy (DPN) is a prevalent and debilitating complication of diabetes, affecting a significant proportion of the diabetic population. Neuromodulation, an emerging therapeutic approach, has shown promise in the management of DPN symptoms. This systematic review aims to synthesize and analyze the current advancements in neuromodulation techniques for the treatment of DPN utilizing studies with preclinical animal models. A comprehensive search was conducted across multiple databases, including PubMed, Scopus, and Web of Science. Inclusion criteria were focused on studies utilizing preclinical animal models for DPN that investigated the efficacy of various neuromodulation techniques, such as spinal cord stimulation, transcranial magnetic stimulation, and peripheral nerve stimulation. The findings suggest that neuromodulation significantly alleviated pain symptoms associated with DPN. Moreover, some studies reported improvements in nerve conduction velocity and reduction in nerve damage. The mechanisms underlying these effects appeared to involve modulation of pain pathways and enhancement of neurotrophic factors. However, the review also highlights the variability in methodology and stimulation parameters across studies, highlighting the need for standardization in future research. Additionally, while the results are promising, the translation of these findings from animal models to human clinical practice requires careful consideration. This review concludes that neuromodulation presents a potentially effective therapeutic strategy for DPN, but further research is necessary to optimize protocols and understand the underlying molecular mechanisms. It also emphasizes the importance of bridging the gap between preclinical findings and clinical applications to improve the management of DPN in diabetic patients.

## Introduction

Diabetic Peripheral Neuropathy (DPN) is a debilitating complication of diabetes, impacting a substantial population across the globe [1–4]. It is primarily characterized by nerve damage resulting from prolonged and cumulative exposure to hyperglycemia [5–7]. The pathophysiology of DPN involves a complex interplay of metabolic and vascular factors driven by prolonged hyperglycemia, leading to nerve ischemia and subsequent neuronal injury [8–11]. The clinical manifestation of DPN is diverse, presenting a range of symptoms that significantly impair the quality of life of affected individuals [12–14]. Patients commonly experience sensory abnormalities including pain, tingling, burning sensations, and numbness, predominantly in the lower extremities [15]. These sensory deficits not only cause discomfort and pain but also contribute to a loss of protective sensation. As a result, individuals with DPN are at an increased risk of developing foot ulcers and infections, which in severe cases can lead to limb amputations [16–20]. Additionally, DPN can manifest as motor and autonomic dysfunction, further complicating the clinical picture [21]. Although there are pharmaceutical interventions for the treatment of DPN, many patients remain dissatisfied with their treatment [22–24]. This has led to the pursuit of new treatment modalities, including neuromodulation.

Preclinical studies using animal models are crucial in the field of biomedical research, especially for the development and refinement of novel therapies. Their importance is particularly significant in the study and treatment of complex conditions such as DPN [25, 26]. These models allow for the exploration of disease mechanisms by using approaches that may not be possible in humans due to ethical and practical constraints [27]. Moreover, they enable the study of disease progression and therapeutic interventions over a relatively short time frame, providing rapid insights that can inform clinical research and treatment approaches [28–31]. In the specific context of DPN, animal models serve as a platform for investigating the efficacy of neuromodulatory therapies.

Preclinical animal models have been instrumental in advancing our understanding and treatment of DPN [25]. Several landmark studies have utilized rodent models to uncover the pathophysiological mechanisms of DPN, identifying oxidative stress and mitochondrial dysfunction as critical factors in nerve damage [32–38]. Additionally, animal models have been employed to reveal the detrimental effects of both hypoglycemia and hyperglycemia on peripheral nerves [39–43], paving the way for the development of new pharmacological interventions. These key studies besides others highlight the pivotal role of preclinical models in elucidating the complex mechanisms underlying DPN and in the discovery and testing of potential treatment modalities including neuromodulation, highlighting their indispensable value in biomedical research.

**Fig 1:**
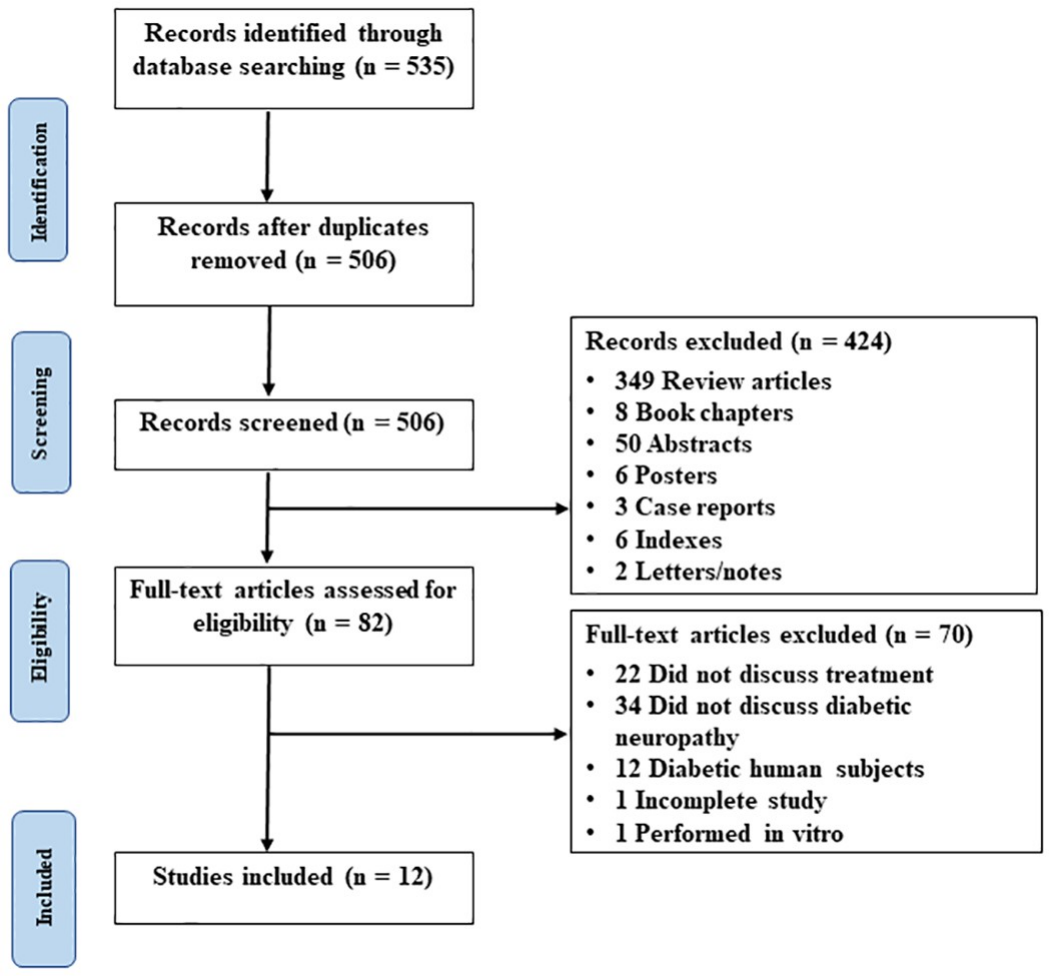
PRISMA flow chart. This figure represents the PRISMA (Preferred Reporting Items for Systematic Reviews and Meta-Analyses) flow diagram detailing the process of study selection for our systematic review.

**Fig 2:**
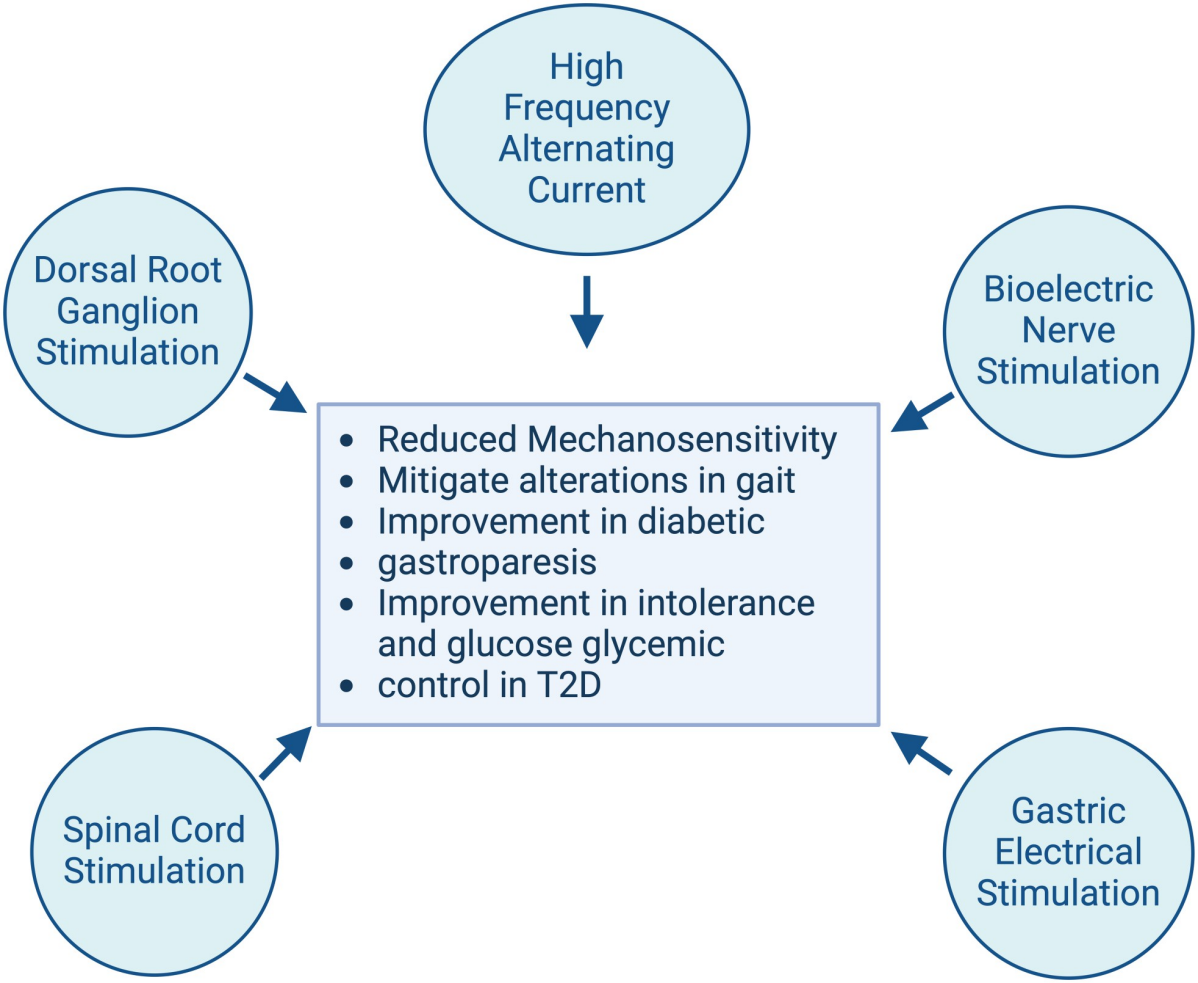
A topographical representation of diabetic peripheral neuropathy (DPN) symptom relief utilizing various neuromodulation techniques in preclinical animal models.

Neuromodulation represents a cutting-edge therapeutic approach that utilizes electrical or chemical stimulation to relieve pain and improve nerve function [44–46]. This systematic review aims to discuss current advancements regarding the use of neuromodulation for the treatment of DPN in preclinical animal models (Fig 1). Neuromodulation encompasses a variety of techniques. Dorsal root ganglion stimulation (DRGS) and spinal cord stimulation (SCS) provide electrical stimulation to the spinal cord. DRGS transmits the impulses directly to the area of the spinal cord responsible for transmitting sensory and pain signals. High frequency alternating current (HFAC) is utilized to block nerve conduction. Bioelectric nerve stimulation utilizes electrical stimulation of nerve fibers to decrease transmission of pain signals to the brain. Finally, gastric electrical stimulation provides electrical impulses to the stomach to stimulate contraction and increase gastric emptying (Fig 2). These techniques offer novel ways to alleviate neuropathic pain and improve nerve function by directly altering nerve activity [47–60].

**Fig 3:**
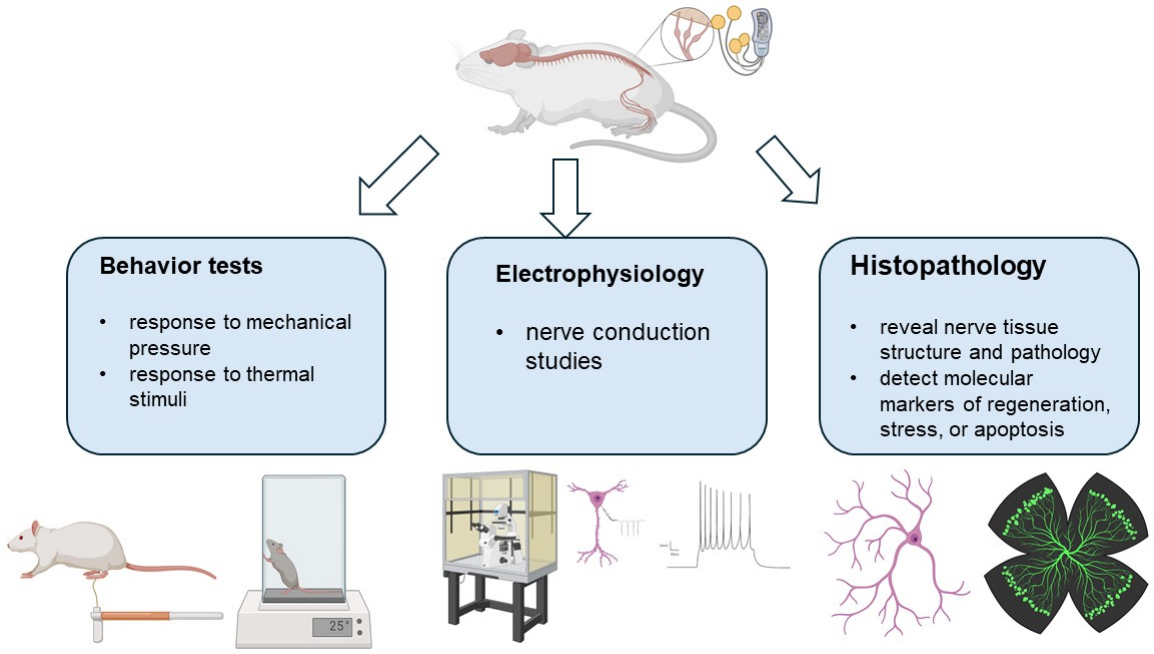
A schematic representation of behavior tests utilized in preclinical animal models for assessing the efficacy of diabetic peripheral neuropathy (DPN). Created with BioRender.com.

**Fig 4:**
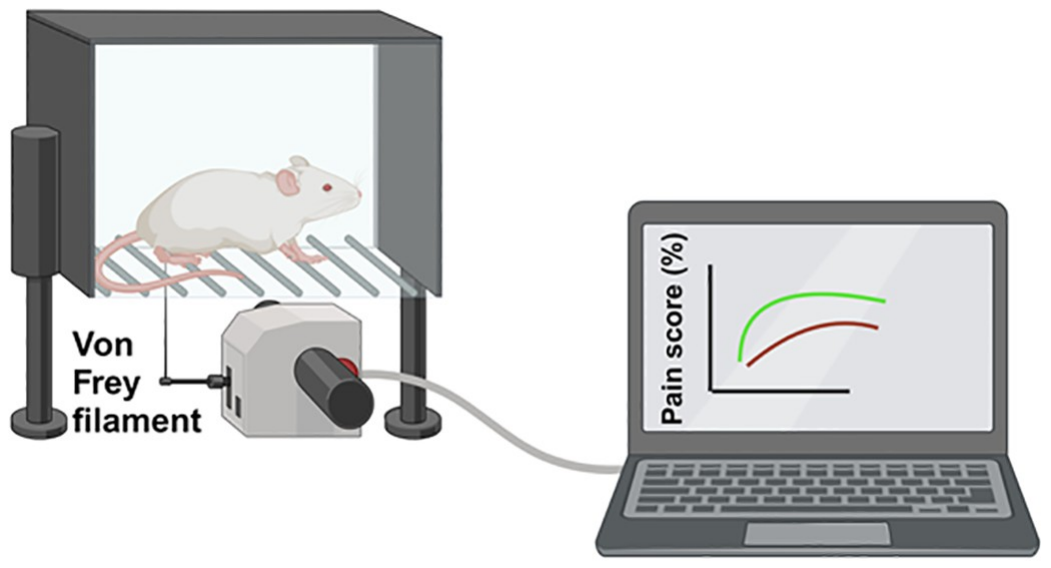
The Von Frey test in mechanical pain sensitivity assessment. This figure displays the essential components of the Von Frey test apparatus used to measure mechanical pain thresholds in preclinical animal models of diabetic peripheral neuropathy (DPN). The setup includes a series of calibrated Von Frey filaments of varying forces, a testing platform with a mesh bottom that allows for unobstructed access to the plantar surface of the animal, and a containment unit to restrict the movement of the animal while permitting natural posture. Each filament is applied perpendicularly to the plantar surface of the hind paw with sufficient force to cause a slight bend in the filament, and the response to each filament in terms of pain score is recorded. Created with BioRender.com.

To assess the effectiveness of neuromodulatory strategies in animal models, a variety of tests have been utilized that can broadly be categorized into several groups. One of the primary types of assessments used in these models is behavioral testing (Fig 3) [61]. These tests are designed to evaluate the sensory and pain responses in animals, which can be indicative of the neuropathic pain experienced by humans with DPN. For instance, the hot plate test measures the response to thermal stimuli, while the von Frey filament test (Fig 4) assesses the response to mechanical pressure [62–65]. The changes in response after neuromodulatory treatment can suggest an improvement in the symptoms of DPN. Electrophysiological assessments are another critical component of the evaluation process (Fig 3) [66]. These tests, which include nerve conduction studies, provide direct measurements of nerve function [67–69]. They can help determine whether neuromodulatory interventions restore or improve the electrical signaling in nerves damaged by diabetes, which is directly relevant to the symptoms of DPN. In addition, biomarker analysis can also provide critical information regarding the efficacy of neuromodulation for DPN [70, 71]. By measuring specific biomarkers in animal models, it is possible to gain insight into the molecular changes that occur in response to neuromodulation. Markers of inflammation, nerve damage, or regeneration (such as proinflammatory cytokines and growth factors) may offer an understanding of the biochemical impact of the neuromodulation, while histological examinations offer a more structural perspective for observing the physical changes in nerve tissue following neuromodulatory treatments (Fig 3) [72, 73]. These can reveal the extent of nerve damage or repair, thus confirming the efficacy of neuromodulation. Finally, motor function tests such as the rotarod test can evaluate the impact of DPN and its treatment on the motor abilities of the animal [74–77]. Since DPN often affects muscle strength and coordination, improvements in these areas can be a strong indicator of treatment success.

While preclinical animal models have significantly advanced our understanding of DPN, several limitations and challenges must be acknowledged to provide a balanced perspective. One major limitation is the potential for species-specific differences that may not fully replicate human disease pathology, leading to translational gaps when applying findings to clinical settings. Methodological issues, such as variability in animal handling, housing conditions, and experimental protocols, can introduce biases and inconsistencies in results. Additionally, many studies often rely on small sample sizes and lack rigorous controls, which can undermine the statistical power and reproducibility of findings. There is also a tendency to publish positive results, potentially skewing the literature and overlooking unsuccessful or negative studies that could provide valuable insights. Furthermore, while animal models can mimic certain aspects of DPN, they may not capture the full complexity of the disease as it occurs in humans, particularly regarding chronic disease progression and comorbidities. These limitations highlight the need for improved standardization, larger and more diverse study cohorts, and the integration of complementary research methods to enhance the validity and applicability of preclinical findings to human DPN research.

This systematic review aims to explore experimental neuromodulation in DPN through preclinical animal models, examining its potential to offer deeper insights into effective therapies for this debilitating condition. The studies performed using these models have produced encouraging outcomes, suggesting that neuromodulation holds significant promise for alleviating the painful symptoms of DPN (Fig 2) [78–80].

## Methods

### Search strategy

This study was performed in compliance with the Preferred Reporting Items for Systematic Reviews and Meta-Analyses (PRISMA) guidelines, and its robustness was further augmented by adhering to the recommendations outlined in the Cochrane Collaboration Handbook. Before starting the study, a detailed protocol was created and registered in the PROSPERO database (registration number: CRD42023490688). The literature searches were performed between January 1, 2018, to October 1, 2023, as narrative or systematic review articles were available covering studies up to 2017. We searched PubMed (MEDLINE), Science Direct, Scopus, and EMBASE databases utilizing the following MeSH terms: ("Neuromodulation"[Mesh]) AND ("Diabetic Neuropathy"[Mesh]) AND ("Animal Models"[Mesh]) AND (“Preclinical Studies"[Mesh]). Where MeSH search was not available, the following Boolean terms were used ("Neuromodulation") AND ("Diabetic Neuropathy") AND ("Animal Models") AND (“Preclinical Studies").

### Inclusion and exclusion criteria

Studies that included animal models with diabetes-induced neuropathy, confirmed through established diagnostic methods such as diminished vibration sense using a tuning fork, abnormal monofilament pressure sensation, decreased reflexes, and reduced pinprick sensitivity, were selected for inclusion. Exclusion criteria were applied to studies lacking confirmed diabetes-induced neuropathy in animal models, as well as to review articles, meta-analyses, abstract-only publications, conference proceedings, editorials/letters, case reports, or articles published before January 1, 2018. Case reports were excluded to ensure the inclusion of more robust and generalizable evidence in our systematic review. Case reports often lack control groups and may not provide sufficient evidence for broader applicability. Furthermore, studies conducted *in vitro* or *ex vivo* were also excluded as this would not allow for evaluation of behavioral changes. *In vitro* or *ex vivo* studies, while valuable for mechanistic insights, do not replicate the complex interactions present *in vivo*.

The process of screening involved an independent review of titles, abstracts, and full texts by at least two trained reviewers (K.M., G.K., E.M., R.M.). Any disagreements regarding inclusion or exclusion were resolved through a consensus among the reviewers or by consulting with other researchers participating in this study. The initial screening was based on titles and abstracts, followed by a detailed full-text analysis for further assessment.

### Data extraction

The data was extracted by at least two trained reviewers (K.M., G.K., E.M., R.M.) and selected in accordance with an appropriate experimental model. Results were grouped together based on neuromodulation technique.

### Quality and risk of bias assessment

Assessment of the quality of each study included in our review was independently performed by at least two reviewers (K.M., G.K., E.M., R.M.) utilizing the Systematic Review Centre for Laboratory Animal Experimentation (SYRCLE) risk of bias (RoB) tool [81]. Any disagreements were resolved by consensus between the reviewers or discussion with other investigators of this study. SYRCLE is an adaptation of the Cochrane’s RoB tool for clinical studies, tailored specifically for animal research. It comprises a checklist of ten items: 1) was the allocation sequence adequately generated and applied?, 2) were the groups similar at baseline or were they adjusted for confounders in the analysis?, 3) was the allocation to the different groups adequately concealed?, 4) were the animals randomly housed during the experiment?, 5) were the caregivers and/or investigators blinded from knowledge of which intervention each animal received?, 6) were animals selected at random for outcome assessment?, 7) was the outcome assessor blind?, 8) were incomplete outcome data adequately addressed?, 9) are reports of the study free of selective outcome reporting?, and 10) was the study apparently free of other problems that could result in high risk of bias? The answers were “Yes” if the study adequately addressed the item, “No” if it did not, or “Unclear” if there was insufficient information for a definitive answer. These responses then informed categorization of the risk of bias domains as either low, high, or unclear. Subsequently, an overall risk of bias assessment was determined and recorded for each study.

## Results

A total of 534 studies were retrieved using the predefined search algorithm as described in the methods section. After deduplication, 505 studies were included for title and abstract screening. After screening, 424 studies were excluded based on irrelevance and 81 articles were included for whole-text analysis. A total of 69 articles were then excluded as 22 did not discuss a treatment, 34 did not discuss diabetic neuropathy, 12 were not performed on preclinical models, 1 was an incomplete study, and 1 was performed *in vitro*. Finally, 12 articles remained for inclusion in the literature review and qualitative analysis.

**Fig 5:**
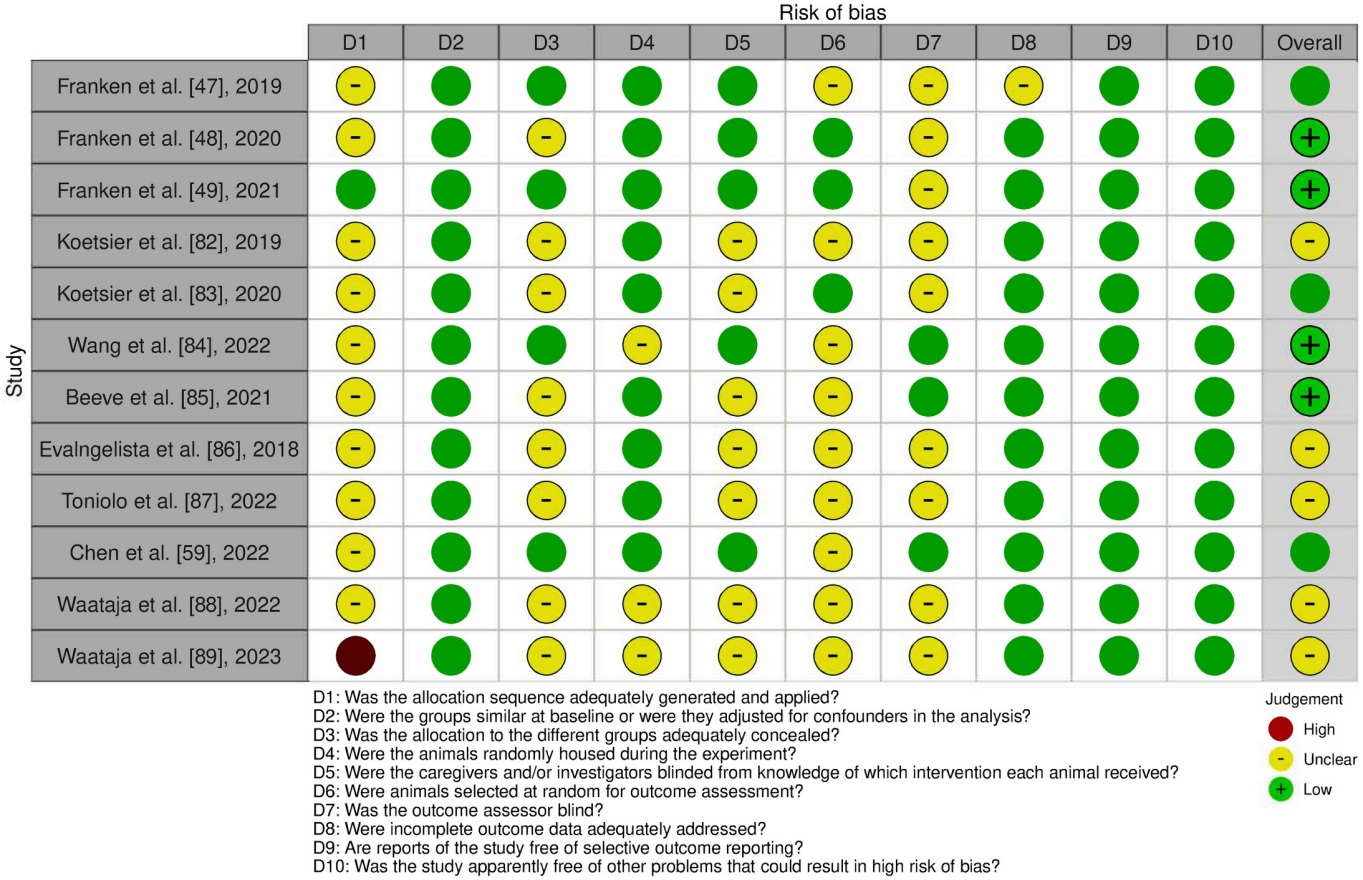
Risk of bias (RoB) assessment utilizing the Systematic Review Centre for Laboratory Animal Experimentation (SYRCLE).

A total of 12 articles published between 2018 and 2023 were included in this systematic review shown in the PRISMA diagram (Fig 1). Approximately 250 animals were collectively evaluated across the included articles. Of the 12 studies, nine evaluated dorsal root ganglion stimulation (DRGS) and three evaluated other unique techniques. Most of the studies were trials where animals were diabetes-induced and randomized to receive neuromodulatory treatment. The search strategy employed for the included studies is shown in the PRISMA diagram in Fig 1 and Risk of Bias is outlined in Fig 5. Seven of the 12 studies were found to have a low risk of bias, 5 a moderate risk of bias, and 0 a high risk of bias. Bias was mostly introduced in these studies due to lack of appropriate information on randomization, blinding, and allocation sequence. Overall, however, the studies were determined to be of appropriate quality to be included in the review. A summary of each study design, patient grouping, and included results is presented in Table 1.

**Table 1:** A summary of included studies.

Reference	Study	Population	Exposure	Comparative Group	Outcomes
Franken et al. [47], 2019	Animal model	22 female Sprague-Dawley rats with STZ induced diabetes, tested for mechanical hypersensitivity 4 weeks after induction	Randomized burst or conventional DRGS at L5 (n = 10)	Sham DRGS (n = 7)	Significant reduction in mechanical hypersensitivity in the conventional DRGS group at 15 minutes (p<0.05) and 30 minutes (p<0.01), which returned to baseline after 45 minutes post treatment Significant reduction in mechanical hypersensitivity in the burst DRGS group at 15 minutes (p<0.01) and 30 minutes (p<0.02), which returned to baseline after 45 minutes post treatment No significant difference in values when comparing burst vs conventional DRGS groups
Franken et al. [48], 2020	Animal model	9 female Sprague-Dawley rats with STZ induced diabetes, tested for mechanical hypersensitivity 4 weeks after induction	Burst DRGS at 0%, 10%, 33%, 50%, 66%, and 80% of motor threshold	Baseline mechanical hyper-sensitivity	Mechanical hypersensitivity was found to be significantly reduced at 15, 30, and 45 minutes compared to baseline at 33%, 50%, 66%, and 80% (p < 0.05) No significant reduction was found at 0% and 10% Burst DRGS at 50% was found to be significantly more effective in normalizing the hypersensitivity when compared to stimulation at 80% (p <0.05) Optimal DRGS amplitude of 54% at 15 minutes and 51% at 30 minutes. Overall optimal DRGS amplitude of 52%
Franken et al. [49], 2021	Animal model	14 female Sprague-Dawley rats with STZ induced diabetes, tested for mechanical hypersensitivity 4 weeks after induction	Conventional DRGS at L5 (n = 8)	Sham DRGS (n = 6)	Conventional DRGS significantly attenuated mechanical hypersensitivity, while no significant difference was found for the sham treatment group Anti-GABA immune-stained dorsal root ganglion revealed strong intracellular reactivity, suggesting that conventional DRGS operates in a GABA-independent mechanism
Koetsier et al. [82], 2019	Animal model	18 female Sprague-Dawley rats with STZ induced diabetes, tested for mechanical hypersensitivity 4 weeks after induction	Conventional DRGS (n = 11)	Sham DRGS (n = 7) SCS group from previous study	The SCS group showed a significant improvement at 15 minutes (p = <0.05) but an insignificant improvement at 30 minutes (p = 0.11) No differences were observed between the 15- and 30-minute time points (p = 0.69) The DRGS group showed a significant improvement at both 15 and 30 minutes (p <0.01 for both) There was a significant improvement between the 15- and 30-minute time points (p <0.05) The washout effect was significantly lower for the DRGS group than that of SCS (p <0.05)
Koetsier et al. [83], 2020	Animal model	10 female Sprague-Dawley rats with STZ induced diabetes, tested for mechanical hypersensitivity 4 weeks after induction	30 minutes of 1Hz, 20Hz, and 1000Hz frequency DRGS during 4 days	Sham DRGS	When compared to Sham group 15 minutes post-treatment, the 20Hz and 1000Hz produced significant results (p<0.001 and p<0.0001 respectively) while the 1Hz was not significant (p = 0.07) When compared to the Sham group 30 minutes post treatment, all three frequencies showed improvement (p<0.001 in all) After cessation of 1 Hz treatment, values did not return to baseline at 45 or 60 minutes (p <0.001 and p <0.01, respectively) For the 20 Hz treatment, values did not return to baseline after 45 minutes but did after 60 minutes (p <0.05 and p = 0.29, respectively) For the 1000 Hz treatment, values returned immediately to baseline at 45 minutes and remained at baseline at 60 minutes (p = 0.06 and p = 0.26, respectively)
Wang et al. [84], 2022	Animal model	29 male Sprague-Dawley rats	STZ induced diabetic rats treated with 10 kHz SCS	Naïve control rats STZ induced Diabetic control rats. STZ induced Diabetic Sham treatment rats.	Behavioral thresholds via Von Frey filaments were significantly higher in naïve (p <0.0001) and STZ+10kHzSCS (p = 0.03) compared to STZ+ShamSCS The mean receptive field area in the dorsal horn of the L5-L6 segments was significantly greater for the STZ+ShamSCS group than for STZ+10kHzSCS (p = 0.0126) and naïve (p <0.001) Mechanosensitivity with innocuous brush showed a significantly reduced firing rate in STZ+10kHzSCS compared to STZ+ShamSCS (p = 0.02) STZ+10kHzSCS also demonstrated a reduced firing rate in response to painful pinch compared to STZ+ShamSCS (p = 0.002)
Beeve et al. [85], 2021	Animal model	Male 14-week-old Lewis rats with STZ induced T1DM	Bioelectric Nerve Stimulation (eStim) 0.2ms pulse at 20Hz frequency 1 hour weekly for 8 weeks, started 2 weeks after STZ induction	Non-diabetic control / Sham treatment Non-diabetic control / eStim treatment STZ diabetic / Sham treatment	T1D independently resulted in bilateral reduction of foot-base-angle (FBA) by -12%. This was further reduced unilaterally on the cuffed side in non-stimulated animals by -12 +/- 10% in controls and by -13 +/- 14% in diabetics In the control and diabetic eStim groups, the cuffed limb was partially restored to -3 +/- 7% and -3 +/- 13%, respectively In terms of mechanical allodynia, it was found that the diabetic rats demonstrated approximately a 56% reduction in response threshold bilaterally compared to controls (p = 0.0026). eStim did not alter this sensitivity (p = 0.6957)
Evangelista et al. [86], 2018	Animal model	C57B1/6 mice with STZ induced diabetes	Mesenchymal stem/stromal cell treatment (MSC)	5 Groups: Non diabetic control group STZ induction + control treatment STZ induction + MSC STZ induction + conditioned medium-MSC STZ induction + control medium	Mice treated with MSC demonstrated complete reversal of mechanical allodynia 3 weeks post-treatment (p < 0.001). Late loss of mechanical sensitivity, representing sensory neuropathy, was completely prevented in this group. Additionally, heat hypoalgesia was reverted (p < 0.001) When observing the sciatic nerve, it was found that diabetic mice had significant morphologic alterations compared to controls. However, these abnormalities were completely reversed in mice treated with MSC (p < 0.05). Similar results were demonstrated when evaluating the unmyelinated C fibers Glial cell expression in the spinal cord was found to be significantly reduced in MSC treated mice compared to untreated diabetic mice (p <0.05) MSC treatment reduced spinal cord mRNA expression of catalase, superoxide dismutase, glutathione peroxidase, and Nrf2 compared to untreated diabetic mice MSC-treated mice had reduced levels of IL-1B and TNF-a and enhanced levels of IL-10 and TGF-B (p < 0.05) MSC treatment also completely reversed the enhanced spinal immunoreactivity for galectin-3
Toniolo et al. [87], 2022	Animal model	Male C57BL/6 mice	STZ induced diabetes	Non-STZ induced controls	Cannabinoid type 1 (CB1R), delta opioid (DOR), and mu opioid receptor (MOR) levels were increased in the diabetic mice compared to saline-treated controls (p <0.001, p <0.01, and p <0.0001, respectively) The CB1R agonist, Hu-210, showed a significant increase in efficacy in the diabetic mice compared to controls The MOR agonist, DAMGO, showed an increase in both potency and efficacy The DOR agonist, deltorphin II, led to an increase in potency In the presence of Hu-210 and hemopressin, another CB1R agonist, DAMGO signaling was increased in diabetic mice (p <0.0001 and p <0.05, respectively) Diabetic mice show a significant increase in the number of CB1R-MOR heteromers (p <0.05), but there were no significant changes in the number of CB1R-DOR heteromers After oral treatment for 14 days, it was found that hemopressin attenuates mechanical allodynia in the diabetic mice compared to the control Treatment with hemopressin does not affect the number of CB1R-MOR heteromers in DOR knockout mice. Conversely, treatment with hemopressin does significantly increase the number of CB1R-DOR heteromers in MOR knockout mice (p <0.05)
Chen et al. [59], 2022	Animal model	Sixty 6–8-week-old Sprague-Dawley rats	Gastric Electrical Stimulation	6 Groups:++Normal non-diabetic control++STZ induced diabetic group++STZ induced diabetic group with sham GES 3 groups of STZ induced diabetes receiving GES at different settings	Interstitial Cells of Cajal (ICC), 5-HT2B receptor expression, 5-HT levels, nNOS neurons, CHAT neurons, and GDNF protein expression were all decreased in the diabetic and sham GES groups compared to control (p-values: <0.008, <0.006, <0.001, 0.002, <0.001, <0.001, respectively) All of these same parameters were increased in GES groups compared to the diabetic rats (p-values: <0.015, <0.006, <0.001, <0.002, <0.002, <0.001, respectively)
Waataja et al. [88], 2022	Animal model	ZDF rats Alloxan treated swine	HFAC	IVGTT and OGTT compared to sham and pre-implantation	ZDF rats had significant improvement in IVGTT during HFAC + stimulation, compared to sham Hepatic vagotomy or celiac stimulation showed no difference, compared to sham Alloxan swine showed significant improvement in OGTT and decrease in FPG with HFAC + stimulation Insulin responses were similar before and after treatment
Waataja et al. [89], 2023	Animal model	Swine with Alloxan induced T2DM or Pre-Diabetes	HFAC	Baseline OGTT and FPG	Performance on the OGTT was improved in both diabetic and pre-diabetic groups (p = 0.003 and p = 0.004, respectively) Significant improvement in FPG levels in both diabetic and pre-diabetic groups (p <0.01) to a non-diabetic level following 2 days of application There was no change in weight in the animals over the course of the experiment

The exploration of neuromodulation for treating DPN in preclinical animal models is an emerging and promising area of research. This approach is being investigated to understand the effects of nerve stimulation techniques on DPN symptoms. Animal models offer a valuable platform for testing various neuromodulation methods, such as spinal cord stimulation and vagus nerve stimulation, to assess their efficacy and safety. These studies are crucial for developing a deeper understanding of neuromodulation’s potential as a novel treatment option for DPN in humans, paving the way for innovative and more effective therapeutic strategies.

### Dorsal root ganglion stimulation (DRGS) for painful peripheral neuropathy

Franken et al. utilized female Sprague-Dawley rats to compare burst DRGS and conventional DRGS in the treatment of PDPN [47]. Diabetes was induced using streptozotocin (STZ) in 48 rats. The rats were tested for mechanical hypersensitivity using the von Frey test both before and four weeks after STZ treatment. A total of twenty-two rats were ultimately selected and implanted at the L5 DRG with a unilateral bipolar lead. Ten of these animals were randomized to receive either burst or conventional stimulation, and seven received sham DRGS. For the conventional group, there was a significant reduction in mechanical hypersensitivity at 15 and 30 minutes (p<0.05 and p<0.01, respectively). Scores returned to baseline 45 and 60 minutes after the treatment was discontinued, with no evidence of residual benefit (p>0.99 at both 45 and 60 minutes). For the burst group, this also resulted in a significant reduction at 15 and 30 minutes (p = 0.01 and p = 0.02, respectively). Baseline values were also re-established at 45 and 60 minutes (p = 0.40 and p = 0.15, respectively). However, there seemed to be residual effect after termination of the treatment as there was no significant difference in the efficacy at 30 minutes compared to 45 minutes. For the sham group, no significant difference was found at any time point. Significant differences were found when comparing conventional vs. sham and burst vs. sham, but there was no difference found when comparing conventional vs. burst. This demonstrates that both conventional and burst are successful in decreasing mechanical hypersensitivity caused by PDPN, with burst even showing a residual effect [47].

Franken et al. performed a subsequent study to further evaluate the effects of burst DRGS in the treatment of PDPN [48]. Similar methods were utilized as before. Thirteen rats were implanted and nine ultimately received burst DRGS at 0%, 10%, 33%, 50%, 66%, and 80% of motor threshold in a randomized crossover design. Mechanical hypersensitivity was found to be significantly reduced at 15, 30, and 45 minutes compared to baseline at 33%, 50%, 66%, and 80% (p<0.05). No significant reduction was found at 0% and 10%. Additionally, burst DRGS at 50% was found to be significantly more effective in normalizing the hypersensitivity when compared to stimulation at 80% (p<0.05). The researchers then calculated the optimal DRGS amplitude depending on the time point. This was found to be 54% at 15 minutes and 51% at 30 minutes. They also calculated the overall optimal DRGS, which was found to be 52%. This helps to better understand the non-linear relationship between burst DRGS and behavioral outcome when treating PDPN [48].

Franken et al. performed an additional study to evaluate the mechanism by which conventional DRGS produces a reduction in pain [49]. It has been hypothesized that this is through a GABA-dependent mechanism. Again, similar methods were utilized as their previous studies. Fourteen animals were ultimately implanted with the device. Eight received conventional DRGS and six received sham DRGS. The animals’ DRG were then immunostained for GABA. As demonstrated in their previous studies, conventional DRGS was found to significantly attenuate the mechanical hypersensitivity, while no significant difference was found for the sham group (p<0.05). The anti-GABA immunostained DRGS showed a very strong intracellular reactivity. No significant differences were found between the groups. This demonstrates that conventional DRGS must be operating in a GABA-independent mechanism since intracellular GABA remains high post-treatment [49].

In addition to the studies performed by Franken et al., Koetsier et al. performed a study using female Sprague-Dawley rats to compare conventional DRGS and SCS in their ability to provide pain relief in PDPN [82]. Diabetes was induced in 48 rats using STZ. Eighteen rats were ultimately implanted; eleven were assigned to the DRGS group and seven to the sham group. Mechanical hypersensitivity was assessed using the von Frey test. Results were compared to the results of a previous study with similar methods that randomized rats into SCS and sham SCS groups. The SCS group showed a significant improvement at 15 minutes but an insignificant improvement at 30 minutes (p<0.05 and p = 0.11, respectively). No differences were observed between the 15- and 30-minute time points (p = 0.69). The DRGS group showed a significant improvement at both 15 and 30 minutes (p<0.01 for both). Additionally, there was a significant improvement between the 15- and 30-minute time points (p<0.05). However, the washout effect was significantly lower than that of SCS (p<0.05). Neither sham therapy resulted in significant improvements. This demonstrates that both SCS and DRGS provide a significant reduction in mechanical hypersensitivity, but DRGS seems to washout more quickly [82].

Koetsier et al. performed a subsequent study to evaluate the pain-relieving effect of DRGS at varying frequencies [83]. Methods were similar to their previous study. Ten rats were ultimately implanted and received treatment. The study was a randomized crossover design, so each animal received alternating 30 minutes of 1 Hz, 20 Hz, and 1000 Hz frequencies, as well as sham DRGS, for four days. Von Frey filaments were used to assess mechanical hypersensitivity. The researchers found that all frequencies produced significant improvements in mechanical hypersensitivity at both 15 and 30 minutes (1 Hz: p<0.01 and p<0.0001; 20 Hz: p<0.0001 and p<0.0001; 1000 Hz: p<0.0001 and p<0.0001). Interestingly, they found a difference in the washout periods between the three frequencies. After cessation of 1 Hz treatment, values did not return to baseline at 45 or 60 minutes (p<0.001 and p<0.01, respectively). For the 20 Hz treatment, values did not return to baseline after 45 minutes but did after 60 minutes (p<0.05 and p = 0.29, respectively). Finally, for the 1000 Hz treatment, values returned immediately to baseline at 45 minutes and remained at baseline at 60 minutes (p = 0.06 and p = 0.26, respectively). When compared to the sham group, only the 20 Hz and 1000 Hz frequencies produced significant results at 15 minutes (1 Hz: p = 0.07; 20 Hz: p<0.001; 1000 Hz: p<0.0001). However, all three frequencies showed significant improvement compared to the sham group at 30 minutes (1 Hz: p<0.001; 20 Hz: p<0.001; 1000 Hz: p<0.0001). These results suggest that all three frequencies are equally effective in reducing symptoms of PDPN, but 1 Hz may be the most optimal due to its delayed washout period compared to the other two frequencies [83].

### Other neuromodulatory treatments for painful peripheral neuropathy

In addition to DRGS, other treatment options that have been identified including SCS, bioelectric nerve stimulation, mesenchymal stem/stromal cells, and modulation of cannabinoid/opiod receptors.

First, Wang et al. performed a study to better understand the effects of low-intensity 10 kHz SCS on PDPN in a rat model [84]. Diabetes was induced in 25 male Sprague-Dawley rats using STZ. Twelve of the STZ-induced rats were then implanted. Eight of the implanted rats received 10 kHz SCS and four received sham treatment. Behavioral assays were performed using von Frey filaments. They found that thresholds were significantly higher in naïve (p<0.0001) and STZ+10kHzSCS (p = 0.03) compared to STZ+ShamSCS. STZ-Control was similar to that of STZ+ShamSCS. Similarly, before treatment, thresholds were significantly greater for naïve (p = 0.007) and STZ+10kHzSCS (p = 0.02) compared to STZ+ShamSCS but were not significantly different from STZ-Control. They also assessed several electrophysiological measures. First, they found the mean receptive field area in the dorsal horn of the L5-L6 segments to be significantly greater for the STZ+ShamSCS group than for STZ+10kHzSCS (p = 0.0126) and naïve (p<0.001). Next, mechanosensitivity with innocuous brush showed a significantly reduced firing rate in STZ+10kHzSCS compared to STZ+ShamSCS (p = 0.02). STZ+10kHzSCS also demonstrated a reduced firing rate in response to painful pinch (p = 0.002). These improvements in the treatment group, compared to the sham group, demonstrate that this technique may be a useful option for the treatment of PDPN [84].

Additionally, Beeve et al. performed a study on STZ-induced rats (T1D) to address the efficacy of another potential treatment option, bioelectric nerve stimulation (eStim), in preventing the onset of PDPN [85]. Rats were randomized into four groups: control/sham, control/eStim, T1D/sham, T1D/eStim. eStim devices were implanted unilaterally onto the right sciatic nerve. Stimulation groups received treatment under anesthesia 1 hour weekly for 8 weeks. The sham group also underwent general anesthesia for 1 hour weekly but did not receive the treatment. Gait analysis was performed pre- and post-treatment by measuring the foot-to-base angle (FBA) of each foot while the animal was actively walking. Mechanical allodynia was measured using von Frey monofilaments. The researchers found that T1D independently resulted in bilateral reduction of FBA by 12%. This was further reduced unilaterally on the cuffed side in non-stimulated animals by 12 ± 10% in controls and by 13 ± 14% in diabetics. However, in the control and diabetic eStim groups, the cuffed limb was partially restored to -3 ± 7% and -3 ± 13%, respectively. In terms of mechanical allodynia, it was found that the diabetic rats demonstrated approximately a 56% reduction in response threshold bilaterally compared to controls (p = 0.0026). This suggests an increased sensitivity to mechanical stimuli, and eStim did not alter this sensitivity (p = 0.695). This demonstrates that stimulation may help to mitigate alterations in gait but does not seem to have a significant impact on sensory alterations secondary to PDPN [85].

Next, Evangelista et al. conducted a study to understand the possible therapeutic effect that mesenchymal stem/stromal cells (MSC) have on spinal neuroinflammation and peripheral nerve regeneration in diabetic peripheral neuropathy [86]. The study was performed on STZ-induced C57B1/6 mice. Mice were divided into five groups: control, STZ + control treatment, STZ + MSC, STZ + conditioned medium-MSC, and STZ + control medium. The von Frey and Hargreaves tests were used to evaluate mechanical and nociceptive thresholds, respectively. Light microscopy and transmission electron microscopy were utilized to analyze the sciatic nerve. Markers of inflammation were studied using radioimmunoassay, real-time PCR, and immunofluorescence. Mice treated with MSC demonstrated complete reversal of mechanical allodynia 3 weeks post-treatment (p<0.001). Late loss of mechanical sensitivity, representing sensory neuropathy, was completely prevented in this group. Additionally, heat hypoalgesia was reverted (p<0.001). When observing the sciatic nerve, it was found that diabetic mice had significant morphologic alterations compared to controls. However, these abnormalities were completely reversed in mice treated with MSC (p<0.05). Similar results were demonstrated when evaluating the unmyelinated C fibers. The researchers also evaluated MSC treatment effect on neuroinflammation. Glial cell expression in the spinal cord was found to be significantly reduced in MSC-treated mice compared to untreated diabetic mice (p<0.05). MSC treatment reduced spinal cord mRNA expression of catalase, superoxide dismutase, glutathione peroxidase, and Nrf2 compared to untreated diabetic mice. It was also found that MSC-treated mice had reduced levels of IL-1β and TNF-α and enhanced levels of IL-10 and TGF-β (p<0.05). MSC treatment also completely reversed the enhanced spinal immunoreactivity for galectin-3. These results show that treatment with MSC is able to improve sensory alterations, revert morphologic abnormalities present in the nerves, and decrease inflammatory reactions in mice with diabetes [86].

Finally, Toniolo et al. performed a study to evaluate if interactions between cannabinoid and opioid receptors could be targeted for the treatment of PDPN [87]. The study utilized male STZ-induced C57BL/6 mice. Mechanical allodynia was assessed using von Frey filaments. They found that cannabinoid type 1 (CB1R), delta opioid (DOR), and mu opioid receptor (MOR) levels were increased in the diabetic mice compared to saline-treated controls (p<0.001, p<0.01, and p<0.0001, respectively). Additionally, the CB1R agonist, Hu-210, showed a significant increase in efficacy in the diabetic mice compared to controls. The MOR agonist, DAMGO, showed an increase in both potency and efficacy, while the DOR agonist, deltorphin II, led to an increase in potency. Also, in the presence of Hu-210 and hemopressin, another CB1R agonist, DAMGO signaling was increased in diabetic mice (p<0.0001 and p<0.05, respectively). Deltorphin II signaling was unaffected. They also found that diabetic mice showed a significant increase in the number of CB1R-MOR heteromers (p<0.05), but there were no significant changes in the number of CB1R-DOR heteromers. Next, they assessed whether treatment with hemopressin affected mechanical allodynia in the diabetic mice. After oral treatment for 14 days, it was found that hemopressin attenuated mechanical allodynia in the diabetic mice compared to the control. Finally, they found that treatment with hemopressin did not affect the number of CB1R-MOR heteromers in DOR knockout mice. Conversely, treatment with hemopressin did significantly increase the number of CB1R-DOR heteromers in MOR knockout mice (p<0.05). These results suggest that these three receptors play a role in pain modulation in PDPN, and hemopressin could be a potential therapeutic option [87].

### Treatment of other forms of diabetic neuropathy

In addition to painful peripheral neuropathy, diabetic patients suffer from neuropathy in other parts of the body, leading to gastroparesis or difficulty with glycemic control. Studies have been completed to identify neuromodulatory treatment for these conditions as well.

Chen et al. conducted a study to determine if gastric electrical stimulation (GES) is a plausible treatment of diabetic gastroparesis [59]. This was accomplished by evaluating its effect on the interstitial cells of Cajal (ICC), serotonin receptor expression (5-HT2B), serotonin levels (5-HT), nNOS neurons in the myenteric plexus, CHAT nerves in the myenteric layer, and GDNF protein expression. The experiment was performed on STZ-induced male Sprague-Dawley rats. Forty of the 50 diabetic rats had electrodes implanted into the serosal layer of the greater curvature of the stomach. The rats were split into a total of six groups: normal control, diabetic, diabetic with sham GES, and three groups of diabetic animals with GES at different settings. The researchers found that ICC, 5-HT2B receptor expression, 5-HT levels, nNOS neurons, CHAT neurons, and GDNF protein expression were all decreased in the diabetic and sham GES groups compared to control (p-values: <0.008, <0.006, <0.001, 0.002, <0.001, <0.001, respectively). However, they found that all of these same parameters were increased in GES groups compared to the diabetic rats (p-values: <0.015, <0.006, <0.001, <0.002, <0.002, <0.001, respectively). These results demonstrate that GES may help to increase some of the major factors implicated in the pathogenesis of diabetic gastroparesis, perhaps reversing its severity [59].

Additionally, Waataja et al. performed multiple experiments to identify treatments to improve glucose tolerance [88]. First, they conducted an experiment with Zucker obese male rats under four conditions: sham, vagotomy with stimulation, positive control vagotomy, and stimulation. Comparisons were only made between the experimental group and sham group. One hour after these procedures were completed, an intravenous glucose tolerance test (IVGTT) was administered. Plasma glucose levels were raised by 63 ± 12% 5 minutes post-injection in the sham group and remained elevated for 30 minutes with partial recovery (AUC = 1543 ± 257 AU). The hepatic vagotomy and stimulation groups showed no significant difference in area under the curve (AUC) compared to the sham group (vagotomy AUC = 1425 ± 157 AU, stimulation AUC 1220 ± 250 AU). There was a significant decrease in AUC when comparing the sham group to the vagotomy + stimulation group (AUC = 618 ± 111 AU, p<0.01). HFAC was applied 15 minutes before and during a 30-minute IVGTT. Following the IVGTT, there was a significant decrease in AUC compared to the sham group (AUC = 898 ± 68, p <0.05). Fifteen minutes post HFAC + stimulation, a second glucose injection induced a large increase in AUC, which was non-significant. Sprague Dawley rats demonstrated a similar and significant pattern to the ZDF rats. When the celiac branch was stimulated with either concurrent hepatic litigation or concurrent delivery of 5000 Hz, there was a significant decrease in glucose compared to the sham group (sham = 1704 ± 553 AU, vagotomy + stimulation AUC = 202 ± 322 AU p < 0.05, HFAC + stimulation AUC = 418 ± 140 AU, p < 0.05) [88].

The next tests were performed through HFAC + stimulation in Alloxan-treated swine [88]. Two Viking cuff electrodes were implanted on the vagal trunks in the Alloxan swine. Ten days later, three oral glucose tolerance tests (OGTT) were conducted, with two days in between. There was no significant difference after the OGTTs compared to pre-implantation OGTTs. The AUC during the HFAC + stimulation OGTTs showed no significant difference, but there was a significant decrease in AU compared to sham (sham = 6228 ± 1293 AU, HFAC + stimulation = 2225 ± 825 AU, p = 0.015). The average fasting plasma glucose (FPG) for the sham group was 120 ± 14 mg/dL, which was consistent over three OGTTs. Following the first HFAC + stimulation, plasma glucose declined to 67 ± 5 mg/dL. The FPG remained decreased at 69 ± 4 mg/dL at the third HFAC + stimulation OGTT experiment. Insulin was also measured. There was a slight but non-significant increase in baseline insulin following HFAC + stimulation experiments (baseline prior to HFAC + stimulation = 10.7 ± 2.6 μIU/ml vs. baseline following HFAC + stimulation tests = 15.4 ± 2.9 μIU/ml, p = 0.28). Insulin increased to a non-significant degree following the HFAC + stimulation experiments [88]. The results from this experiment show that the electrical blockade of the hepatic vagus branch with stimulation of the celiac vagus branch may be a localized approach for type 2 diabetes (T2D) treatment [88].

The final experiment conducted by Waataja et al. assessed whether combined celiac and hepatic vagus nerve neuromodulation could improve glucose intolerance and glycemic control in T2D [89]. T2D was induced in swine models using alloxan. The swine were then separated into a diabetic and a pre-diabetic group depending on their resulting fasting glucose levels. HFAC was applied to the hepatic branches of the vagus in order to block its output, and stimulation was applied to the celiac branch feeding the pancreas. They found that performance on the OGTT was improved in both diabetic and pre-diabetic groups (p = 0.003 and p = 0.004, respectively). There was also a significant improvement in fasting plasma glucose (FPG) levels in both diabetic and pre-diabetic groups (p<0.01), improving to a non-diabetic level following two days of application. There was no change in weight in the animals over the course of the experiment, so results do not seem to be related to weight loss. This may therefore be a viable treatment option to decrease both the development and progression of T2D [89].

## Discussion

The majority of the outcomes from the included studies were encouraging. In five studies, it was observed that DRGS, both burst and conventional, were successful in producing a significant decrease in mechanical hypersensitivity in the animals [47–49, 82, 83]. Furthermore, one study found that burst DRGS seems to have a residual effect compared to conventional DRGS [47]. Also, one study reported that the dose response to burst DRGS is non-linear, observing optimal response at 50% of the motor threshold [48]. Another study found that 1 Hz DRGS may be the most effective due to a delayed washout compared to the other frequencies tested [83]. Across these studies, it seems that burst DRGS and 1 Hz DRGS may produce the longest response.

Finally, it was thought that DRGS operates through a GABA-dependent mechanism. However, one study found that this may not be the case, as intracellular levels of GABA remained high post-treatment [49].

SCS was also found to significantly improve mechanosensitivity in two studies [82, 84]. Furthermore, when compared to conventional DRGS, SCS seemed to have a slower washout [82].

When looking at other methods of treatment, it was found in one study that eStim has a significant effect on gait improvement but does not seem to improve sensory alterations [85]. This is in contrast to DRGS and SCS, which were both seen to improve sensory dysfunction. Also, MSCs were found in one study to cause complete reversal of allodynia and decrease inflammatory markers and reactive glial replication in the spinal cord [86]. Finally, one study found that CB1R, MOR, and DOR play a role in modulating diabetic peripheral neuropathy and that hemopressin may be a potential treatment option to alter this system [87].

In summary, DRGS and SCS demonstrate significant improvements in markers of PDPN and could be viable treatment options. Furthermore, the application of hemopressin and MSC has demonstrated encouraging outcomes and could represent an innovative method for modulating this system. The success of these studies in preclinical settings underscores their potential efficacy. This progress marks a significant step forward in developing more targeted and sophisticated treatment strategies for PDPN, leveraging insights gained from animal models to potentially translate into human clinical applications.

Three additional studies used neuromodulatory techniques to target other forms of diabetic neuropathy, including gastroparesis and nerve dysfunction involved in glycemic control [59, 88, 89]. The first study found that GES in the setting of gastroparesis was found to decrease ICC, 5-HT2B receptor expression, 5-HT levels, nNOS neurons, CHAT neurons, and GDNF protein expression, which are all implicated in the disease progression [59]. Both studies performed by Waataja et al. found that both the OGTT and FPG were improved in animals that received celiac and hepatic vagus nerve stimulation. Of note, the animals’ weights did not change throughout the experiment, so this effect seemed to be weight-independent [88, 89]. Additionally, ZDF rats showed significant improvement in IVGTT compared to sham after HFAC and stimulation [88]. These represent intriguing non-pharmacological approaches to manage such diabetic complications. Additionally, employing these techniques to enhance glucose tolerance could potentially decelerate the progression of the disease, thereby reducing the risk of further neuropathic damage.

### Limitations

One of the primary limitations of the included investigations in this systematic review is the inherent variability in the preclinical animal models used across the studies. Different species, strains, and methods of inducing diabetic neuropathy can lead to significant variations in neuropathic symptoms and responses to neuromodulation therapies. This heterogeneity makes it challenging to generalize the findings to a broader context, including potential applications in human treatments for DPN. The review also encountered limitations due to methodological differences in neuromodulation techniques used in animal models. Differences in neuromodulation techniques, parameters, and outcome measurements can lead to inconsistencies in results and sometimes make it difficult to compare results across various studies.

Another limitation is the lack of long-term efficacy data in the reviewed studies. Most preclinical trials have short follow-up periods. Therefore, the long-term benefits, potential side effects, and sustainability of neuromodulation treatments in animal models of DPN remain largely unexplored. This gap in data limits the ability to draw comprehensive conclusions about the long-term effectiveness and safety of these neuromodulation therapies.

## Conclusions and future directions

This systematic review highlights the potential of various neuromodulation techniques in mitigating the symptoms of DPN in preclinical studies. These techniques, which include spinal cord stimulation, peripheral nerve stimulation, and other emerging modalities, have shown promising results in reducing neuropathic pain and improving nerve function in animal models. The efficacy observed in these studies highlights neuromodulation as a promising avenue for DPN treatment, offering an alternative to conventional pharmacological therapies, which often come with significant side effects. However, further studies are warranted to advance the work being done in this field.

First, there is a need for greater standardization in the animal models used for studying DPN and neuromodulation. The development and adoption of standardized protocols for animal models and neuromodulation techniques will reduce variability and improve the reliability of future studies. Next, future research should focus on long-term studies to evaluate the sustained efficacy and safety of neuromodulation techniques. This will be crucial in understanding the potential chronic impacts and benefits of these therapies. This will also help to determine if these techniques are viable long-term treatment options or if they are only useful in providing short-term relief. This could pontentially be addressed by utilizing animal models with longer life expectancies that could be followed over a longer period of time. Additionally, mechanistic studies are needed to elucidate the underlying pathology through which neuromodulation impacts neuropathic processes in DPN. Understanding these molecular mechanisms will facilitate the optimization of neuromodulation techniques and the development of targeted and personalized therapies. Efforts should also be made to bridge the gap between animal models and human clinical trials. Initiatives that focus on translational research will be vital in determining how the findings from animal studies can be effectively applied in clinical settings to treat DPN in humans. There is a need to develop robust translational research framework for neuromodulation techniques in DPN which should encompass clearly defined steps and methodologies to bridge the gap between preclinical findings and clinical applications. The framework can start with pilot clinical trials designed to validate preclinical results in humans, followed by detailed regulatory considerations to ensure compliance with medical standards and safety protocols. Patient selection criteria must be carefully established to identify suitable candidates based on disease severity, comorbidities, and previous treatment responses. Overcoming translational barriers requires a multi-faceted approach, including thorough cross-species validation of mechanisms, standardized protocols to reduce variability, and collaborative efforts between researchers, clinicians, and regulatory bodies. Detailed proposals on these aspects will ensure that insights from animal models are effectively and safely translated into human treatments. Furthermore, investigating the combined use of neuromodulation with other therapies, such as pharmacological treatments or lifestyle modifications, could provide insight into more comprehensive and effective management strategies for DPN and could potentially lead to synergistic treatment effects.

While further research is warranted, our review suggests that neuromodulation is a promising treatment modality for DPN in pursuit of improving quality of life of affected induviduals.

## Supporting information

S1 Checklist PRISMA 2020 checklist. (DOCX)
